# Predictive modelling of Ross River virus using climate data in the Darling Downs

**DOI:** 10.1017/S0950268823000365

**Published:** 2023-03-14

**Authors:** Julia Meadows, Celia McMichael, Patricia T. Campbell

**Affiliations:** 1 School of Geography, Earth and Atmospheric Sciences, Faculty of Science, The University of Melbourne, 221 Bouverie St, Carlton, VIC 3053, Australia; 2 Department of Infectious Diseases, Melbourne Medical School, University of Melbourne at the Peter Doherty Institute for Infection and Immunity, Melbourne, VIC 3000, Australia; 3 Centre for Epidemiology and Biostatistics, Melbourne School of Population and Global Health, The University of Melbourne, Melbourne, VIC 3010, Australia

**Keywords:** Arboviruses, climate, mosquito-borne disease, negative binomial regression, Ross River virus

## Abstract

Ross River virus (RRV) is the most common mosquito-borne infection in Australia. RRV disease is characterised by joint pain and lethargy, placing a substantial burden on individual patients, the healthcare system and economy. This burden is compounded by a lack of effective treatment or vaccine for the disease. The complex RRV disease ecology cycle includes a number of reservoirs and vectors that inhabit a range of environments and climates across Australia. Climate is known to influence humans, animals and the environment and has previously been shown to be useful to RRV prediction models. We developed a negative binomial regression model to predict monthly RRV case numbers and outbreaks in the Darling Downs region of Queensland, Australia. Human RRV notifications and climate data for the period July 2001 – June 2014 were used for model training. Model predictions were tested using data for July 2014 – June 2019. The final model was moderately effective at predicting RRV case numbers (Pearson's *r* = 0.427) and RRV outbreaks (accuracy = 65%, sensitivity = 59%, specificity = 73%). Our findings show that readily available climate data can provide timely prediction of RRV outbreaks.

## Introduction

Ross River virus (RRV) disease is the most commonly reported arboviral disease in Australia with approximately 5000 cases notified annually [1, 2]. This is likely an underestimate of the true disease burden due to mild and non-specific disease symptoms. It is endemic to Australia and Papua New Guinea with known outbreaks occurring in the Pacific and potential for expansion into new geographic areas [3–6]. Approximately 25–45% of human RRV infections lead to symptomatic disease characterised by fever, rash, lethargy and polyarthritis [7] with the remaining 55–75% of infections resulting in asymptomatic responses. Managing RRV involves significant healthcare costs and economic burden – including healthcare, mosquito management and lost productivity – estimated to be AUD$ 15 million annually [7]. Current RRV treatment is limited to management of symptoms.

The enzootic cycle of RRV alternates through largely macropod reservoir hosts and mosquito vectors, with human infection and disease occurring through spillover events [6, 7]. A range of environmental, social and political factors such as urbanisation, income level and recreational activities are linked to RRV disease ecology via their influence on behaviours, biology and proximity of humans, reservoir hosts and vectors [8, 9]. Environmental factors such as vegetation, water sources, topography and built environments also affect the density and distribution of reservoir hosts and mosquito vectors involved in the transmission cycle [5, 6, 10].

Climatic factors including temperature, rainfall and humidity play a key role in shaping environments and thereby the RRV transmission cycle [4, 5, 11]. Climate is a key determinant of mosquito biology, affecting aspects such as lifespan, reproduction rates, blood feeding and extrinsic viral incubation periods [12, 13].

Climate has been a major focus of RRV research, particularly for predicting disease outbreaks and cases [14–19]. The delay between climate and mosquito biological and behavioural responses can be exploited to develop predictive models. Early warning systems for climate-sensitive diseases, such as RRV, are identified as a key climate change adaptation strategy [19]. The delay built into predictive models allows time for relevant public health action to reduce the size and impact of the outbreak [20]. These public health actions could include public health messaging to encourage mosquito avoidance behaviour such as use of insect repellents and protective clothing as well as staying indoors [1]. Mosquito control programs using larvicide in mosquito habitats could also be used to reduce the impact of predicted outbreaks [21].

In this study, we focused on the effect of climate and environment on RRV because of their strong influence on mosquito vectors. We excluded entomological and human socioeconomic data to examine the predictive capacity of readily available, open-access climate data alone. While most Australian research on the relationship between climate and RRV has occurred in coastal regions [16–19, 22], we focused on an inland region. We investigated the predictive power of climatic and environmental data for RRV disease in the Darling Downs, Queensland.

## Methods

### Study area

**Fig. 1.**
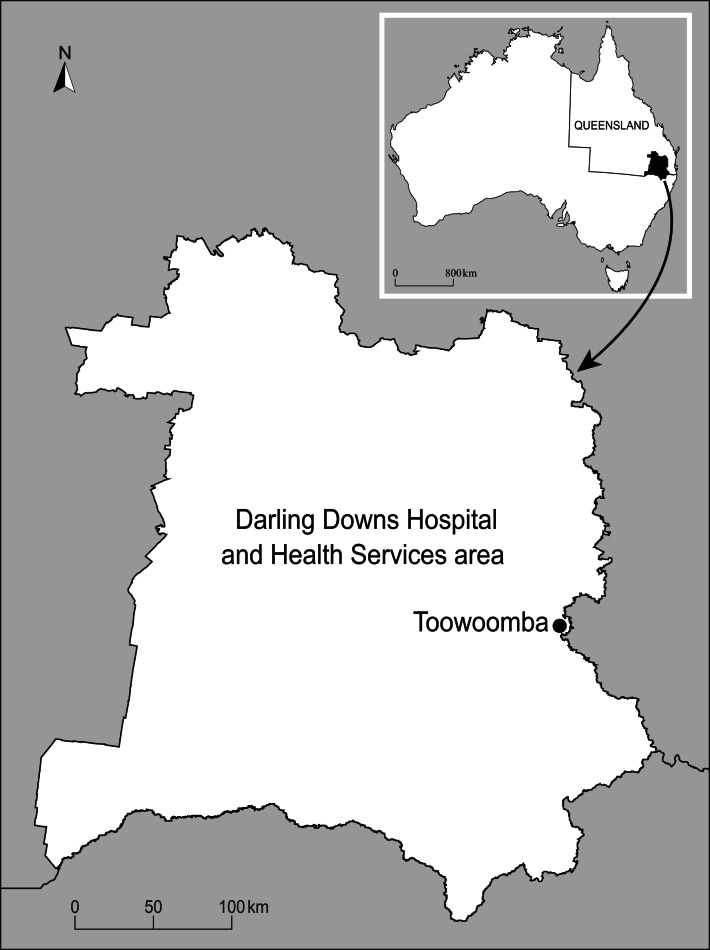
Map of the Darling Downs region.

The Darling Downs (Fig. 1) is located in south-eastern Queensland with a population of 300 000 people [23]. The mostly agricultural region is characterised by a temperate climate with hot summers and cool winters [24]. It was chosen due to high case numbers inland location and small geographic area. High case numbers ensure that investigating the disease in the area will have a useful public health impact. Moreover, most studies predicting RRV have occurred in coastal regions [14]. Inland areas are likely to experience different dynamics of RRV due to different mosquito species and environments. Finally, the small area of the Darling Downs meant that climate was likely to be consistent across the region and a single weather station could be used to collect climate data.

### Statistical methods

**Fig. 2.**
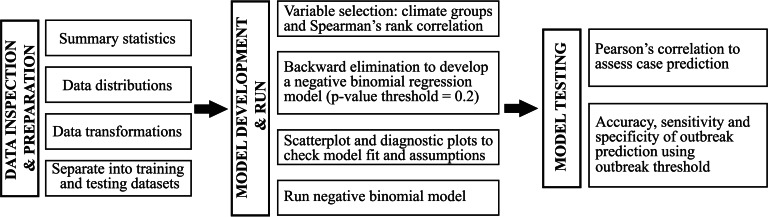
Flowchart of methods.

We implemented a modified version of the statistical methods of Cutcher *et al*. [15] and Koolhof *et al*. [14]. This included, as outlined in Figure 2, the use of data transformations to normalise the distribution of climate variables, the variable selection process using climate groups, inspection of different time lags, use of negative binomial regression and testing the model performance using outbreak thresholds and Pearson's correlation. Ethics approval was not required because only publicly accessible, aggregated data were used in the analysis.

Aggregated monthly case notification data (RRV data) were obtained from the Queensland Department of Health from July 2001 to June 2019 for the Darling Downs Hospital and Health Services area. Years were defined from July to June to capture a single mosquito breeding season per year [15]. The proportion of infections and subsequent disease notified to government databases has remained consistently low overtime due to high proportions of mild and/or non-specific symptoms. However, there have been slight alterations to RRV disease case definitions over the study period. In 2013, more specific notification criteria were developed to clearly distinguish cases of RRV disease from Barmah Forest virus. Further, in 2016, the use of a single IgM to diagnose RRV was removed and the case definition was expanded to include both confirmed and probable cases. In order to account for changes in population size overtime, mid-year population data were sourced from the Australian Bureau of Statistics (2001–2011) and the Health of Queenslanders (2014, 2016, 2018), with derived averages used for years with no population data available.

All climate and weather data were publicly available Australian Bureau of Meteorology data (listed in Supplementary Table 1) obtained from the Toowoomba Airport (041529) weather station as the station nearest to the largest population centre in the Darling Downs with the most complete dataset. Monthly and daily climate data were obtained from January 2001 to June 2019. Daily climate data were used to derive other monthly climate variables such as number of days per month above or below a certain level of precipitation or temperature. Climate data were included up to six months before notification data to account for the delayed effects that climatic conditions can have on the RRV disease ecology cycle through incubation periods (extrinsic and intrinsic), delays between infection and symptom onset and indirect biological delays on the mosquito lifecycle.

A negative binomial regression model was chosen because it is well suited to the positive skew and overdispersion of RRV data [14, 15, 20, 25]. The model used lagged monthly climate data as independent variables, monthly notified RRV data as the dependent variable and yearly population data from the Darling Downs as an offset value to account for variations in population size [25–28]. A flowchart of methods is shown in Figure 2.

First, the RRV data and all climate variables were examined using summary statistics and plotting distributions. Where possible, climate variables with non-normal distributions were transformed so that they resembled an approximately normal distribution [14] (Supplementary Table 1). Approximately normal distributions are useful for analysis of seasonally driven variables because they allow for seasonal variables to be treated with stationarity [14, 15].

The transformed climate data were merged with the RRV data and separated into a 13-year ‘training dataset’ (July 2001–June 2014) and a 5-year ‘testing dataset’ (July 2014–June 2019). The training dataset was used to develop the predictive negative binomial model. Climate and population data from the testing dataset were used to run the predictive model and observed RRV data were compared to the predicted RRV cases.

Spearman's rank correlation (*r*_s_) was used to determine climate variables strongly associated with RRV cases occurring one to six months later and identify the time lag at which the strongest association occurred [14–16]. These correlations were useful for model development: they identify variables which were most useful for prediction, but also reveal potentially important associations between climate and RRV [14–16].

Potential predictors were sorted into nine groups measuring similar climatic aspects (henceforth referred to as ‘climate groups’): temperature, precipitation, relative humidity, vapour pressure, evaporation, evapotranspiration, solar radiation, mean sea level pressure and Southern Oscillation Index (Supplementary Table 1). The variable/lag combination with the strongest association with RRV data was selected from each climate group [14, 15]. To ensure minimal correlation between predictor variables, a requirement of negative binomial regression [25], Spearman's rank correlation matrix was used to control and check for collinearity between the variables selected from each climate group [14–16]. When correlation was strong (defined as |*r*_s_| > 0.8) between any two variables, the variable with the strongest association with RRV was retained [14, 15]. This occurred progressively until a set of variables with the strongest association with RRV data and low correlation to each other remained for use in model development. Backward-stepwise selection was used to develop the predictive negative binomial model with a threshold *P*-value of 0.2 [14, 15, 29].

The model fit was inspected using Pearson's correlation (*r*) and a scatterplot of model-fitted and observed values. Diagnostic plots of scaled residuals (difference between observed and model-predicted values) were run to check that model assumptions (such as distribution and dispersion) were met [15, 30, 31] (Supplementary Figures 1a & 1b).

Model performance was tested by comparing model-predicted RRV cases with observed RRV cases from the testing dataset using Pearson's correlation [14, 15]. The ability of the model to predict monthly outbreaks was determined through the creation of a moving outbreak threshold to which observed and predicted case numbers were compared [14, 15]. We defined an outbreak month as a month with RRV cases exceeding the 5-year rolling mean plus one standard deviation for that month, with known outbreak years excluded from the calculation [14, 15]. This definition captures the epidemiological definition of outbreaks which refers to an increase in disease in excess of what is normally expected in a given time, population and area [15, 32]. The purpose of the threshold was to determine if the model could predict the occurrence and timing of outbreaks. Capturing the magnitude of outbreaks is unlikely given the large variation between baseline and outbreak case numbers used to train the model and because climate variables only act as indirect predictors of disease transmission [15]. A moving threshold was chosen to account for the impact of seasonal variations and long-term climate change on disease transmission [14, 15]. The threshold was calculated using RRV case notification data for the entire study period. RRV data for outbreak years were substituted with RRV data from the previous year. For the first five years of the dataset, the outbreak threshold was calculated using cumulative means and standard deviations.

This threshold was used to identify the number of true positives, true negatives, false positives and false negatives based on comparing model predicted outbreaks and non-outbreaks with observed outbreaks and non-outbreaks. We evaluated the overall model effectiveness and model accuracy (% of months correctly identified), specificity (% of non-outbreak months correctly identified) and sensitivity (% of outbreak months correctly identified). High specificity and sensitivity help avoid adverse consequences from not taking action when needed (low sensitivity) or financial costs from unnecessary action (low specificity).

## Results

There were 2071 RRV notifications over the study period in the Darling Downs. Outbreak years were experienced in 2003/2004, 2005/2006 and 2014/2015 with 283, 257 and 247 cases respectively. Cases were notified year-round, but March had the highest monthly average RRV notifications while August had the lowest.

**Fig. 3.**
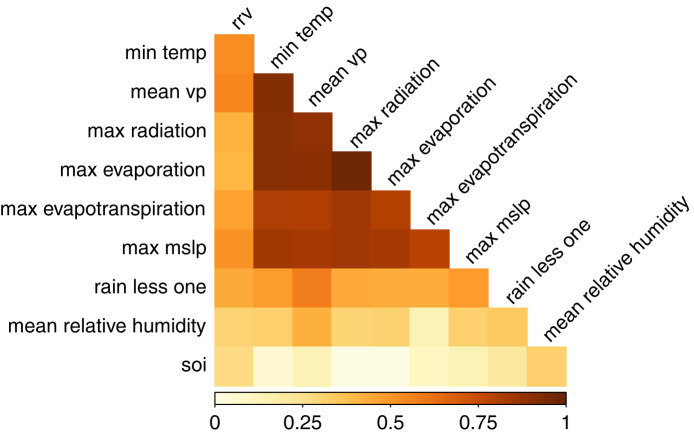
Spearman's correlation matrix: RRV and climate data. min temp, minimum temperature; mean vp, mean vapour pressure; max radiation, maximum solar radiation; max evaporation, maximum Morton's shallow lake evaporation; max evapotranspiration, maximum Morton's areal actual evapotranspiration; max mslp, maximum mean sea level pressure; mean relative humidity, mean relative humidity at maximum daily temperature; rain less one, number of days with less than 1 mm of precipitation; mean relative humidity, mean relative humidity at maximum daily temperature and soi, Southern Oscillation Index.

Monthly RRV data were most strongly correlated with climate at a one-, two- or three-month lag (Supplementary Table 2). Seven climate variables had strong correlations (|*r*_s_| > 0.50) with RRV data: vapour pressure (mean and maximum), temperature (minimum, mean minimum and numbers of days with minimum temperature below 15 °C) and sea level pressure (maximum and mean). RRV was weakly to moderately correlated (0.20 ⩽ |*r*_s_| ⩽ 0.50) with 48 of the 59 climate variables examined. Only five variables had very weak or negligible correlation with RRV at all lags. Despite the large number of variables strongly correlated with monthly RRV data, the correlation matrix (Fig. 3) showed that many of these climate variables were strongly correlated with each other. This resulted in the removal of five variables from the pool of variables considered in the predictive model.

The final model consisted of the Southern Oscillation Index (1-month lag), number of days per month with less than 1 mm of rainfall (2-month lag), mean vapour pressure (2-month lag) and mean relative humidity at maximum daily temperature (1-month lag) as well as a population offset term (log of the population size) (Table 1). Diagnostic plots of the final model (Supplementary Figures 1a & 1b) indicate that there were significant deviations from the uniformity and dispersion assumptions of the negative binomial regression.

**Table 1.** Negative binomial regression model

Independent variable	Lag (months)	Coefficient	IRR (95% CI)	*P*-value	Relationship
Southern Oscillation Index	2	0.02	1.02 (1.00, 1.03)	0.03	Positive
Number of days with less than 1 mm of precipitation	1	−0.03	0.97 (0.92, 1.02)	0.16	Negative
Mean vapour pressure	1	2.94	18.92 (8.39, 43.55)	<0.001	Positive
Mean relative humidity at maximum daily temperature	2	−0.02	0.98 (0.96, 1.01)	0.18	Negative

Coefficients, incidence rate ratios (IRR) and corresponding 95% confidence intervals and *P*-values for independent variables included in final negative binomial regression model.

**Fig. 4.**
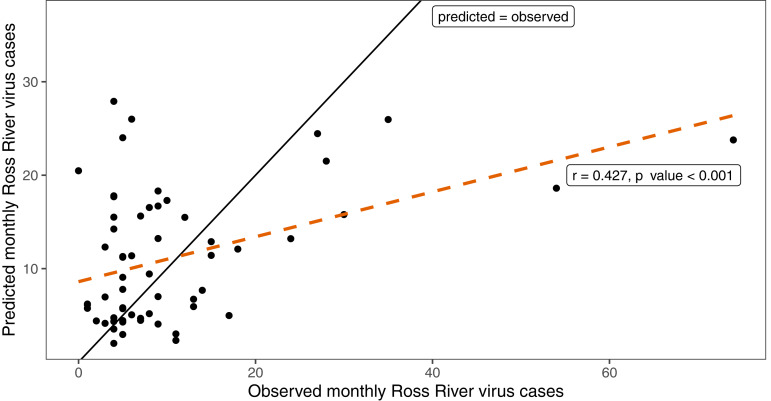
Scatterplot of observed vs predicted monthly RRV case numbers. Observed and model-predicted monthly RRV case numbers from July 2014 to June 2019. The plot illustrates a moderate correlation between observed and model-predicted case numbers. The Pearson's correlation line (*r* = 0.427) is orange and dashed; the black line shows the theoretical line of 100% correlation (predicted = observed).

**Fig. 5.**
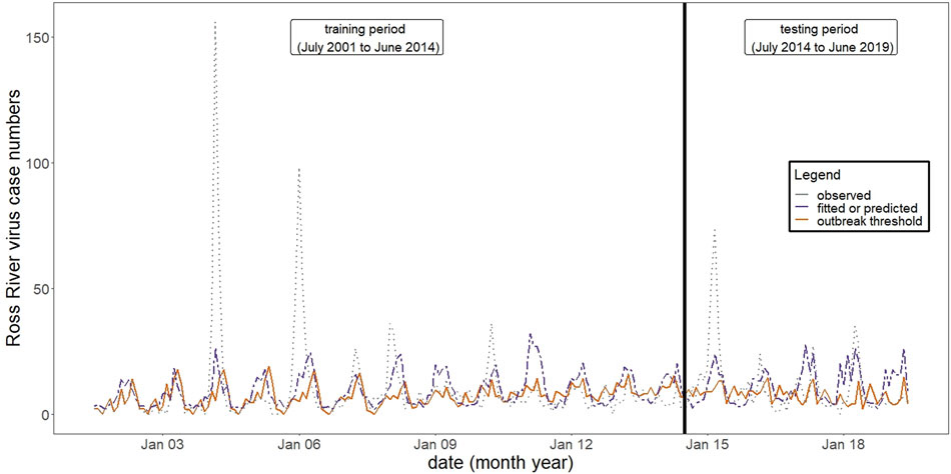
Time-series plot of observed and predicted monthly RRV case numbers. Time series plot showing model fitted (training period) and predicted (testing period) (purple) and observed (training and testing period) (grey) monthly Ross River virus case numbers as well as the moving outbreak threshold (orange). The black vertical line represents the division between training and testing periods. The plot highlights the seasonality of Ross River virus and three large outbreaks which occurred in 2003/2004, 2005/2006 and 2014/2015.

Overall, the model was moderately effective at predicting RRV case numbers, with a Pearson's correlation (*r* = 0.427, 95% CI 0.195–0.615, *P*-value <0.001) between predicted and observed monthly RRV case numbers for the period of July 2014 to June 2019 (Fig. 4). In addition, the model was also moderately effective at predicting the occurrence (not magnitude) of monthly RRV outbreaks and non-outbreaks. The observed and predicted monthly RRV case numbers as well as the calculated outbreak threshold are shown in Figure 5. In 39 of 60 test months (accuracy of 65%), the predicted outcome (outbreak or non-outbreak month) matched the observed outcome (outbreak or non-outbreak month). The model had 59% sensitivity and 73% specificity (see Table 2).

**Table 2.** Confusion matrix values

		Observed	
		Outbreak month	Non-outbreak month
Predicted	Outbreak month	True positive (TP) 20	False positive (FP) 14
	Non-outbreak month	False negative (FN) 7	True negative (TN) 19

True positives, false negatives, false positives and true negatives displayed in the table are used to calculate accuracy, sensitivity and specificity. Accuracy = (TP + TN)/ (TP + TN + FP + FN). Sensitivity rate = TP/(TP + FN). Specificity rate = TN/(TN + FP).

## Discussion

This study is one of only a few to investigate whether climatic and environmental variables are useful predictors of RRV cases and outbreaks in an inland region [15, 26, 27]. The model was moderately effective at predicting monthly RRV case numbers and outbreaks in the Darling Downs, consistent with the role that climate and environment play in the disease ecology cycle and transmission of RRV. In the final model, which included the Southern Oscillation index and variables measuring precipitation, vapour pressure and relative humidity, predicted cases moderately correlated with observed case numbers over the testing period (*r* = 0.427). In addition, the model was moderately successful at predicting outbreak and non-outbreak months with an accuracy of 65%, sensitivity of 59% and specificity of 73%. This means the model is slightly skewed towards false positives instead of false negatives and may sometimes indicate that public health action is needed when it is not. Direct inclusion of factors such as reservoir and vector population dynamics may improve model efficacy if such data were available.

All of the climate variables included in the final negative binomial regression model are similar to those included in previous RRV research [10, 14–16, 18, 19, 26, 27, 33–35]. For example, many variables related to vapour pressure were strongly associated with RRV cases and monthly mean vapour pressure was present in the final predictive model. In particular, higher mean vapour pressure was strongly associated with, and used to predict, higher RRV case numbers occurring two months later. This result is supported by previous research including Cutcher *et al*. [15] which also included vapour pressure as a key predictor of RRV cases in the inland Victorian region of Mildura. Vapour pressure was also present in models from Woodruff *et al*. [35] and Koolhof *et al*. [14]. The importance of vapour pressure, as it measures atmospheric water content, is linked to humidity which greatly influences mosquito biology and behaviour [14, 15]. Increased humidity leads to increased blood-feeding frequency and longevity, increasing the chance of RRV transmission between mosquitos, humans and reservoir hosts [3, 12].

Despite the link between vapour pressure and humidity, both mean vapour pressure and mean relative humidity at maximum daily temperature were included in the final model because the collinearity was not sufficient to need to drop one of variables (*ρ*_s_ = 0.427, *P*-value <0.001). This may be due to the different optimal time lags selected for each variable (2 months for mean vapour pressure and 1 month for relative humidity). However, it is also probable that the key difference between the two measures may explain the relevance and inclusion of both within the final RRV model. Vapour pressure is an absolute measure of atmospheric water content while relative humidity takes temperature into account due to its impact on the maximum atmospheric water vapour capacity [36]. Therefore, vapour pressure and relative humidity may have different interactions with RRV disease ecology [3, 12]. The inclusion of both variables in the final model differs to previous RRV research which has considered both vapour pressure and relative humidity and included only one in the final model [14, 15, 35].

Precipitation has also been widely highlighted as a key predictor of RRV incidence and outbreaks [14–16, 19, 26, 37]. In our study, less than 1 mm of total monthly rainfall was associated with low RRV case numbers two months later and was included in the final model for the Darling Downs. This reaffirms the importance of precipitation to RRV transmission via the mosquito lifecycle and has been supported consistently by previous RRV research and modelling in other regions of Australia [11]. For example, a precipitation-related variable was included in six of the eleven models developed to predict RRV cases and outbreaks in a number of Victorian local government areas [14].

The Southern Oscillation Index (SOI) was the final variable included in the negative binomial regression model. SOI was positively associated with RRV occurring one month later in the Darling Downs. This variable is a measure of the El Niño Southern Oscillation (ENSO), the regional coupling of atmospheric and oceanic circulation affecting temperature and precipitation in the region [22, 34, 38]. In Australia, El Niño conditions (negative SOI) lead to cold and dry weather while La Niña (positive SOI) leads to warm and wet weather conditions [22, 34, 38]. Despite this link between ENSO, precipitation and temperature, SOI was not strongly correlated with temperature or precipitation within the Darling Downs. This may be due to the broad and complex influence of ENSO on the Australian climate and subsequently on RRV transmission, which is yet to be fully researched and understood. Nevertheless, previous studies in Australia have also associated La Niña conditions, or positive SOI values, with RRV outbreaks and incidence and used SOI as a predictor variable in modelling [15, 22, 34, 38, 39].

Many other predictive models for RRV have included measures of temperature, evaporation and evapotranspiration [14, 16, 26, 37]. The absence of these variables in our final model may reflect their reduced importance in RRV transmission in the Darling Downs. However, it is more likely that their absence is due to the high level of multicollinearity amongst climate variables, meaning only one variable can be chosen. In fact, variables measuring temperature, evaporation, evapotranspiration and sea level pressure were strongly correlated with RRV in the Darling Downs despite their exclusion from the model.

Temperature, particularly minimum temperature, has been present in a great number of RRV models [16, 19, 27, 35, 39]. Temperature is crucial to mosquito survival with temperatures below or above thermal thresholds leading to mosquito death curtailing RRV transmission [3, 12, 13]. Despite the exclusion of temperature from the final model for the Darling Downs, most temperature variables were strongly correlated with RRV cases. Similarly, temperature is also related to relative humidity and the SOI, both of which were included in our model for the Darling Downs [3].

Another climate group which was strongly negatively associated with RRV in the Darling Downs but excluded from the final model was mean sea level pressure. Prior studies have associated low sea level pressure and low sea surface temperature with high RRV case numbers [14, 15, 33, 35]. In particular, Koolhof *et al*. [14] were the first to include sea level pressure as a variable within RRV predictive models. Though it is unclear how these oceanic variables affect the disease dynamics of an inland region, it is hypothesised that sea level pressure and sea surface temperature are indicators of the broader ENSO phenomenon [34, 38], included in our model through the SOI.

Solar radiation is the only climate data type investigated in this study which has not previously been reported as associated with RRV cases. Our study found that maximum solar radiation in the Darling Downs was moderately correlated with RRV cases. However, maximum solar radiation was also strongly correlated with measures of temperature, evaporation and evapotranspiration. Therefore, the association with RRV cases may reflect its indirect relationship with other climate variables which directly affect RRV, rather than solar radiation having a direct effect on RRV ecology and transmission.

Models developed for other regions of Australia have provided greater accuracy using a variety of methods including negative binomial regression, linear regression, logistic regression and (seasonal) autoregression moving average models [14, 19, 33]. While alternate methods may provide a better predictive model, our findings may indicate a greater complexity of predicting RRV in inland areas. However, our methodological decisions were based on previous research methods, informed by existing RRV research and evaluated after their employment through model testing and diagnostic plots [14–16, 31, 37]. The greatest strength of this model is that it was developed based solely on open-access climate data which is less resource and cost intensive data to collect and analyse. In addition, the Darling Downs, Queensland was selected due to its high case numbers. In choosing an area with high case numbers the final model was more likely to be statistically and practically important. A weakness is that it is difficult for statistical models to capture the magnitude of variability present in the number of RRV cases notified year-to-year and month-to-month. Moreover, the slight changes to the RRV case definition over the study period, may have influenced the consistency and accuracy of the model. Finally, there is potential for outbreak years to have strongly influenced model development.

This research demonstrates that a moderately effective predictive model for RRV case numbers and outbreaks can be developed for an inland region using solely climate data and confirms the importance of climate to RRV prediction. Future research should include a wider array of variables (e.g. sea surface temperature, sea level, river flow, river height) and compare a broad range of analytical methods. In particular, machine learning and artificial intelligence could be used to automate more robust prediction models [40]. Moreover, this research did not examine interactions between climate variables (e.g. optimal rain and temperature conditions) which may be important for mosquito population and RRV disease ecology. Future research should include a pre-planned, disciplined and rigorous exploration of which climate and environment interactions may be useful for predictive modelling. The development of predictive models – that make use of available climatic, environmental, demographic and entomological data – is important in monitoring and managing vector-borne diseases such as RRV in which a capacity to predict outbreaks can support timely population action.

## Data Availability

Ross River virus disease notification data was obtained for the Darling Downs HHS area through submitting a data request form to the Queensland Department of Health. Available at: https://www.health.qld.gov.au/clinical-practice/guidelines-procedures/diseases-infection/surveillance/reports/notifiable/data-request. Climate and environment data were obtained from the Bureau of Meteorology for Toowoomba Airport weather station (041529) using publicly available historical climate data available at: http://www.bom.gov.au/climate/data/. Darling Downs population data was obtained from the Health of Queenslanders reports (available at: https://www.publications.qld.gov.au/dataset/chief-health-officer-reports) and from the Australian Bureau of Statistics (available at: https://www.qgso.qld.gov.au/statistics/theme/population/population-estimates/regions).

## Supplementary material

For supplementary material accompanying this paper visit http://doi.org/10.1017/S0950268823000365. click here to view supplementary material
