# Supplementary material for: Predictive modelling of Ross River virus using climate data in the Darling Downs
Source: Epidemiol Infect. 2023 Mar 14;151:e55. doi: 10.1017/S0950268823000365 (PMC10126892; doi:10.1017/S0950268823000365)
Supplement: Supplementary file 1 [file hygsup.zip › S0950268823000365sup001.docx]

**Epidemiology and Infection**

**Predictive modelling of Ross River virus using climate data in the Darling Downs**

**Author full names and affiliations**

Julia Meadows

Celia McMichael^1^

Patricia T. Campbell^2,3^

1. School of Geography, Earth and Atmospheric Sciences, Faculty of Science, The University of Melbourne, 221 Bouverie St, Carlton, VIC 3053, Australia
2. Department of Infectious Diseases, Melbourne Medical School, University of Melbourne at the Peter Doherty Institute for Infection and Immunity, Melbourne, VIC 3000, Australia
3. Centre for Epidemiology and Biostatistics, Melbourne School of Population and Global Health, The University of Melbourne, Melbourne, VIC 3010, Australia

**Supplementary Material**

***Supplementary Table S1.*** Transformations applied to climate variables

Variables are divided and displayed in climate groups

| **Variable (monthly/per month)** | **Transformation applied** |
| --- | --- |
| **SOUTHERN OSCILLATION INDEX** | |
| Southern Oscillation Index | — |
| **TEMPERATURE** | |
| Maximum temperature | — |
| Minimum temperature | — |
| Mean maximum temperature | — |
| Mean minimum temperature | — |
| Lowest maximum temperature | — |
| Highest minimum temperature | — |
| Number of days with maximum temperature above 35°C | Cube root |
| Number of days with maximum temperature above 30°C | Square root |
| Number of days with maximum temperature above 25°C | — |
| Number of days with maximum temperature above 20°C | — |
| Number of days with maximum temperature above 15°C | — |
| Number of days with minimum temperature below 20°C | — |
| Number of days with minimum temperature below 15°C | — |
| Number of days with minimum temperature below 10°C | Natural logarithm |
| Number of days with minimum temperature below 5°C | Square root |
| **PRECIPITATION** | |
| Total precipitation | Cube root |
| Number of days with less than 1mm of precipitation | — |
| Number of days with more than 2mm of precipitation | Square root |
| Number of days with more than 5mm of precipitation | Natural logarithm |
| Number of days with more than 10mm of precipitation | Natural logarithm |
| **RELATIVE HUMIDITY** | |
| Maximum relative humidity at maximum daily temperature | — |
| Minimum relative humidity at maximum daily temperature | Square root |
| Mean relative humidity at maximum daily temperature | — |
| Maximum relative humidity at minimum daily temperature | — |
| Minimum relative humidity at minimum daily temperature | — |
| Mean relative humidity at minimum daily temperature | — |
| **VAPOUR PRESSURE** | |
| Maximum vapour pressure | Square root |
| Minimum vapour pressure | Inverse square root |
| Mean vapour pressure | Cube root |
| Maximum vapour pressure deficit | Natural logarithm |
| Minimum vapour pressure deficit | Cube root |
| Mean vapour pressure deficit | Cube root |
| **SOLAR RADIATION** | |
| Maximum solar radiation | — |
| Minimum solar radiation | Natural logarithm |
| Mean solar radiation | — |
| **EVAPORATION** | |
| Maximum class A pan evaporation | Square root |
| Minimum class A pan evaporation | Cube root |
| Mean class A pan evaporation | — |
| Maximum Morton’s shallow lake evaporation | — |
| Minimum Morton’s shallow lake evaporation | Cube root |
| Mean Morton’s shallow lake evaporation | — |
| **EVAPOTRANSPIRATION** | |
| Maximum FAO56 short crop evapotranspiration | — |
| Minimum FAO56 short crop evapotranspiration | Natural logarithm |
| Mean FAO56 short crop evapotranspiration | — |
| Maximum ASCE tall crop evapotranspiration | Square root |
| Minimum ASCE tall crop evapotranspiration | Natural logarithm |
| Mean ASCE tall crop evapotranspiration | — |
| Maximum Morton’s areal actual evapotranspiration | — |
| Minimum Morton’s areal actual evapotranspiration | Square root |
| Mean Morton’s areal actual evapotranspiration | — |
| Maximum Morton’s point potential evapotranspiration | Square root |
| Minimum Morton’s point potential evapotranspiration | Natural logarithm |
| Mean Morton’s point potential evapotranspiration | — |
| Maximum Morton’s wet-environment areal potential evapotranspiration | — |
| Minimum Morton’s wet-environment areal potential evapotranspiration | Cube root |
| Mean Morton’s wet-environment areal potential evapotranspiration | — |
| **MEAN SEA LEVEL PRESSURE** | |
| Maximum mean sea level pressure | — |
| Minimum mean sea level pressure | — |
| Mean sea level pressure | — |

***Supplementary Table S2.*** Optimal time lags and Spearman’s rank correlation of climate variables with RRV cases

Variables are divided and displayed in climate groups and the variable retained from each climate group is highlighted in grey.

| **Variable (monthly/per month)** | **Time lag (months)** | **Correlation with RRV** | |
| --- | --- | --- | --- |
|  |  | **rho (**r_s_) | **p-value** |
| **SOUTHERN OSCILLATION INDEX** | | | |
| Southern Oscillation Index | 1 | 0.274 | <0.001 |
| **TEMPERATURE** | | | |
| Minimum temperature | 2 | 0.533 | <0.001 |
| Mean minimum temperature | 2 | 0.529 | <0.001 |
| Number of days with minimum temperature below 15°C | 2 | -0.522 | <0.001 |
| Number of days with minimum temperature below 10°C | 1 | -0.475 | <0.001 |
| Highest minimum temperature | 2 | 0.443 | <0.001 |
| Number of days with minimum temperature below 5°C | 1 | -0.443 | <0.001 |
| Lowest maximum temperature | 1 | 0.440 | <0.001 |
| Number of days with maximum temperature above 25°C | 2 | 0.398 | <0.001 |
| Number of days with maximum temperature above 20°C | 1 | 0.397 | <0.001 |
| Mean maximum temperature | 2 | 0.396 | <0.001 |
| Maximum temperature | 2 | 0.348 | <0.001 |
| Number of days with maximum temperature above 30°C | 3 | 0.337 | <0.001 |
| Number of days with minimum temperature below 20°C | 2 | -0.299 | <0.001 |
| Number of days with maximum temperature above 15°C | 1 | 0.287 | <0.001 |
| Number of days with maximum temperature above 35°C | 3 | 0.221 | <0.001 |
| **PRECIPITATION** | | | |
| Number of days with less than 1mm of precipitation | 2 | -0.443 | <0.001 |
| Number of days with more than 2mm of precipitation | 2 | 0.428 | <0.001 |
| Total precipitation | 2 | 0.412 | <0.001 |
| Number of days with more than 5mm of precipitation | 2 | 0.386 | <0.001 |
| Number of days with more than 10mm of precipitation | 2 | 0.283 | <0.001 |
| **RELATIVE HUMIDITY** | | | |
| Mean relative humidity at maximum daily temperature | 1 | 0.308 | <0.001 |
| Minimum relative humidity at maximum daily temperature | 6 | -0.282 | <0.001 |
| Minimum relative humidity at minimum daily temperature | 1 | 0.269 | <0.001 |
| Mean relative humidity at minimum daily temperature | 6 | -0.235 | <0.001 |
| Maximum relative humidity at maximum daily temperature | 3 | 0.094 | <0.001 |
| **VAPOUR PRESSURE** | | | |
| Mean vapour pressure | 2 | 0.548 | <0.001 |
| Maximum vapour pressure | 2 | 0.512 | <0.001 |
| Minimum vapour pressure | 2 | -0.475 | <0.001 |
| Maximum vapour pressure deficit | 2 | 0.261 | <0.001 |
| Mean vapour pressure deficit | 3 | 0.259 | <0.001 |
| Minimum vapour pressure deficit | 6 | 0.090 | <0.001 |
| **SOLAR RADIATION** | | | |
| Maximum solar radiation | 3 | 0.420 | <0.001 |
| Mean solar radiation | 3 | 0.309 | <0.001 |
| Minimum solar radiation | 2 | 0.184 | <0.001 |
| **EVAPORATION** | | | |
| Maximum Morton’s shallow lake evaporation | 3 | 0.410 | <0.001 |
| Mean Morton’s shallow lake evaporation | 3 | 0.378 | <0.001 |
| Mean class A pan evaporation | 3 | 0.337 | <0.001 |
| Maximum class A pan evaporation | 3 | 0.323 | <0.001 |
| Minimum Morton’s shallow lake evaporation | 2 | 0.301 | <0.001 |
| Minimum class A pan evaporation | 3 | 0.097 | <0.001 |
| **EVAPOTRANSPIRATION** | | | |
| Maximum Morton’s areal actual evapotranspiration | 2 | 0.474 | <0.001 |
| Mean Morton’s areal actual evapotranspiration | 2 | 0.468 | <0.001 |
| Minimum Morton’s areal actual evapotranspiration | 2 | 0.412 | <0.001 |
| Maximum Morton’s wet-environment areal potential evapotranspiration | 3 | 0.407 | <0.001 |
| Mean Morton’s wet-environment areal potential evapotranspiration | 3 | 0.379 | <0.001 |
| Maximum FAO56 short crop evapotranspiration | 3 | 0.357 | <0.001 |
| Mean FAO56 short crop evapotranspiration | 3 | 0.352 | <0.001 |
| Maximum ASCE tall crop evapotranspiration | 4 | 0.328 | <0.001 |
| Maximum Morton’s point potential evapotranspiration | 4 | 0.325 | <0.001 |
| Mean ASCE tall crop evapotranspiration | 3 | 0.322 | <0.001 |
| Mean Morton’s point potential evapotranspiration | 3 | 0.317 | <0.001 |
| Minimum Morton’s wet-environment areal potential evapotranspiration | 2 | 0.305 | <0.001 |
| Minimum FAO56 short crop evapotranspiration | 2 | 0.249 | 0.002 |
| Minimum Morton’s point potential evapotranspiration | 2 | 0.215 | 0.007 |
| Minimum ASCE tall crop evapotranspiration | 2 | 0.190 | 0.02 |
| **MEAN SEA LEVEL PRESSURE** | | | |
| Maximum mean sea level pressure | 2 | -0.517 | <0.001 |
| Mean sea level pressure | 2 | -0.504 | <0.001 |
| Minimum mean sea level pressure | 2 | -0.345 | <0.001 |

** maximum relative humidity at minimum daily temperature was not included because there was extremely limited variation in values: 154/156 (approx. 99%) of the months in the training dataset reported a maximum relative humidity at minimum daily temperature of 100%.


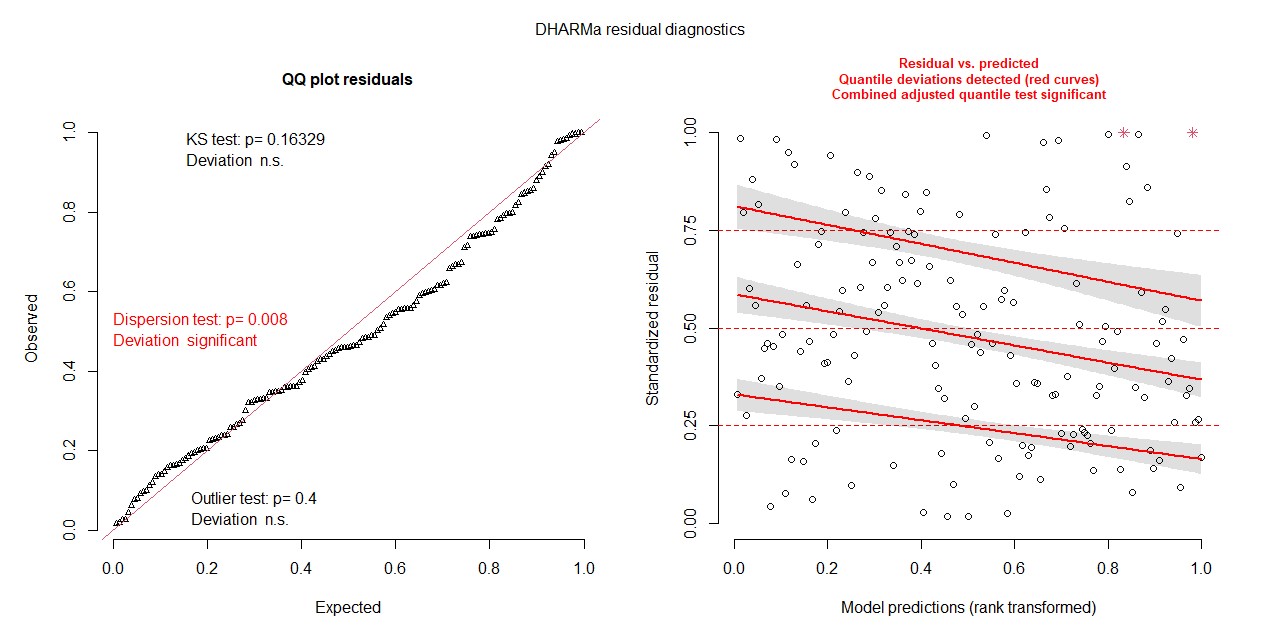


***Supplementary Figures S1a & S1b.*** Diagnostic plots

The DHARMa package in R was used to evaluate the model fit through residual diagnostics (Hartig 2018). The QQ plot was used to test for residuals uniformity, outliers and dispersion. The plot suggests a significant lack of fit (p<0.008) indicating over- or under-dispersion. However, the QQ plot shows that the uniformity and outlier assumptions have been met. The plot showing residuals vs predicted was used to detect deviations from uniformity of residuals and outliers. The plot indicates there is significant deviations from the uniformity assumption.
