# Supplementary figures and images for: Predictive modelling of Ross River virus using climate data in the Darling Downs
Source: Epidemiol Infect. 2023 Mar 14;151:e55. doi: 10.1017/S0950268823000365 (PMC10126892; doi:10.1017/S0950268823000365)

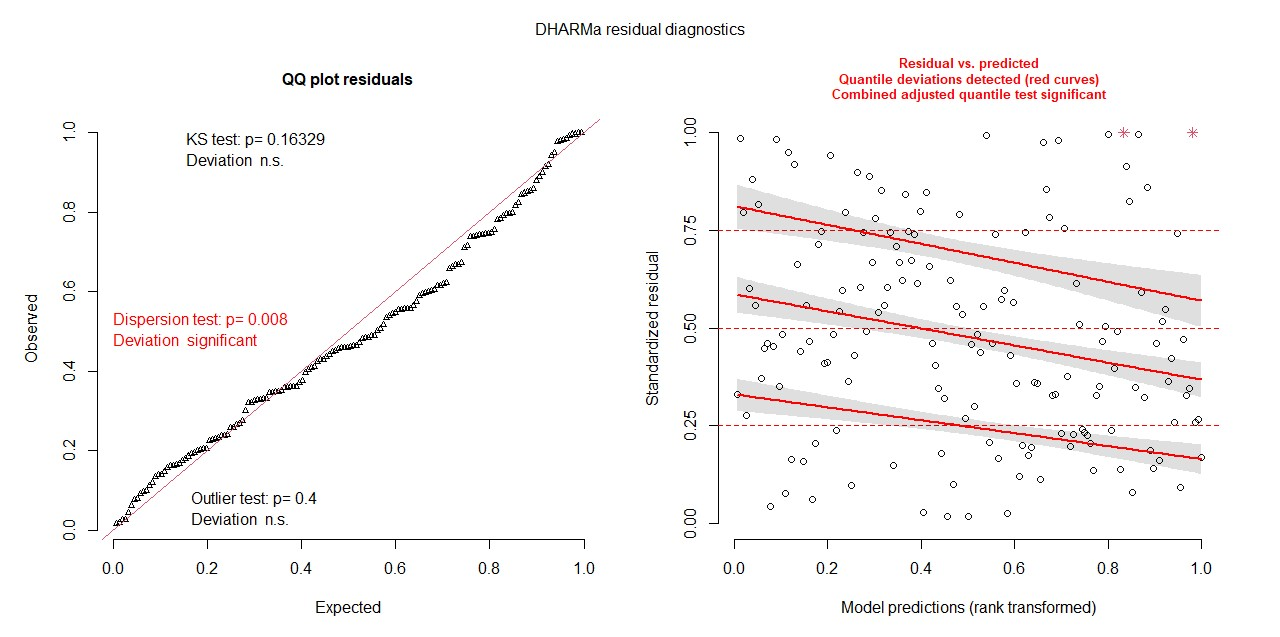

Supplement: Supplementary file 1 [file hygsup.zip › S0950268823000365sup002.tif]
